# Mid term freedom from atrial fibrillation following hybrid ablation, a systematic review and meta analysis

**DOI:** 10.1186/s13019-023-02189-2

**Published:** 2023-04-19

**Authors:** Aditya Eranki, Ashley Wilson-Smith, Campbell Flynn, Michael Williams, Con Manganas

**Affiliations:** 1 Department of Cardiothoracic Surgery, St. George Hospital, Kograh, Sydney, 2217 Australia; 2 The Chris O’Brien Lifehouse Center, Sydney, Australia; 3 The Collaborative Research Group (CORE), Sydney, Australia; 4 The University of Sydney, Sydney, Australia; 5 The John Hunter Hospital, Newcastle, Australia; 6 Department of Cardiothoracic Surgery, Dunedin Hospital, Great King Street, Dunedin Otago, New Zealand

**Keywords:** Hybrid ablation, Convergent procedure, Atrial fibrillation, Freedom from atrial fibrillation

## Abstract

**Introduction:**

Atrial Fibrillation (AF) is a common tachyarrhythmia affecting 33 million people worldwide. Hybrid AF ablation utilises a surgical (epicardial) ablation followed by an endocardial catheter-based ablation. The aim of this systematic review and meta-analysis is to summarize the literature reporting mid-term freedom from AF following hybrid ablation.

**Methods:**

An electronic search of databases was performed to identify all relevant studies providing mid-term (2 year) outcomes following hybrid ablation for AF. The primary study outcome was to assess the mid-term freedom from AF following hybrid ablation, utilising the *metaprop* function on Stata® (Version 17.0, StataCorp, Texas, USA). Subgroup analysis was performed to assess the impact of various operative characteristics on mid-term freedom from AF. The secondary outcomes assessed mortality and procedural complication rate.

**Results:**

The search strategy identified 16 studies qualifying for inclusion in this meta-analysis, with 1242 patients in total. The majority of papers were retrospective cohort studies (15) and one study was a randomized control trial (RCT). The mean follow up was 31.5 ± 8.4 months. Following hybrid ablation, the overall mid-term freedom from AF was 74.6% and 65.4% for patients off antiarrhythmic drugs (AAD). Actuarial freedom from AF was 78.2%, 74.2% and 73.6% at 1, 2 and 3 years respectively. No significant differences in mid-term freedom from AF based epicardial lesion set (box vs pulmonary vein isolation) or Left atrial appendage/Ganglionated Plexus/Ligament of Marshall ablation or staged vs concomitant procedures. There were 12 deaths overall following the hybrid procedure with a pooled complication rate of 5.53%.

**Conclusion:**

Hybrid AF ablation offers promising mid-term freedom from AF reported at a mean follow-up of 31.5 months. The overall complication rate remains low. Further analysis of high-quality studies with randomized data and long-term follow up will help verify these results.

**Supplementary Information:**

The online version contains supplementary material available at 10.1186/s13019-023-02189-2.

## Introduction

Atrial fibrillation (AF) is a common tachyarrhythmia, affecting approximately 33 million people worldwide [1, 2], of which 70% of patients have persistent arrhythmia [2]. Catheter ablation is the mainstay of management in patients with paroxysmal atrial fibrillation (PAF) or persistent atrial fibrillation (PersAF) who are intolerant to class 1 or 3 antiarrhythmic medications and have indication for improved rhythm control (for the purpose of reducing arrhythmia related symptoms or improving left ventricular function) [3]. Repeat catheter ablation procedures are often necessary for sinus rhythm (SR) maintenance with; a systematic review demonstrating a three-year freedom from AF of 53% after a single procedure and 80% following multiple ablations, in a cohort of studies that largely assessed paroxysmal AF [4]. Patients with persistent and longstanding AF often have more resistant substrates and may benefit from surgical ablation [3].

Several surgical approaches to AF ablations have been described of which the Cox-Maze 4 (CM4) has demonstrated the most favourable long-term freedom from AF (at 10 years), of 77% [5, 6]. A class-1 recommendation therefore exists for the CM4 to be performed in the setting of persistent AF or AF resistant to antiarrhythmic drug therapy or failed catheter ablation as a standalone procedure or in patients undergoing concurrent cardiac surgery [3]. However, CM4 is significant undertaking requiring cardiopulmonary bypass meaning standalone CM4 procedures for AF are confined to specialised centres only [7]. With the advent of bipolar radiofrequency clamps and linear ablation devices, a totally thorascopic maze approach is available through a unilateral or bilateral closed chest approach, with a one-year freedom from AF of 82% [8, 9]. One of the major drawbacks of a totally thorascopic approach is the inability to form transmural lesions, which are essential for electrical isolation [9].

Hybrid AF surgery is an evolving field. It comprises of an initial surgical epicardial ablation and second stage transvenous endocardial catheter ablation. This strategy seeks to combine the strengths of both approaches. The epicardial component can be accessed through a thorascopy (unilateral or bilateral) or a laparoscopic subxiphoid approach [10]. In this manner, an antral pulmonary vein isolation lesion set can be applied, a posterior left atrial isolation box lesion set can be deployed (aiming to isolate the left atrial posterior wall *en bloc*). Ablation of the autonomic ganglionic plexi can be performed as well as surgical division of the ligament of Marshall and exclusion of the left atrial appendage [10]. With the advent of electro-anatomical mapping, existing ablation lines can be mapped, thus guiding the second stage endocardial ablation [10]. This can be done in either a staged or concomitant setting [10]. The major strength of this approach is the ability to create epicardial/endocardial ablations which are effectively transmural [10].

Evidence demonstrates the efficacy of hybrid ablation for AF. Varzaly et al. reported a freedom from AF of 79.4% and a complication rate of 6.5%, over a follow up of 19 months [10]. A meta-analysis by Van der Heijden et al. reports a higher freedom from AF following hybrid ablation in patients with longstanding AF, when compared to catheter ablation alone [11]. Studies are now publishing the mid- to long-term freedom from AF following hybrid ablation. The primary outcome of this systematic review and meta-analysis is to assess the mid-term freedom from AF following hybrid ablation for atrial fibrillation. The secondary outcome of this paper is to assess the mortality and complication rate.

## Materials and methods

### Search strategy and study selection

This trial was registered with PROSPERO and was written in accordance with Preferred Reporting Items for Systematic Reviews and Meta-Analyses (PRISMA) recommendations (CRD42022337086). An electronic literature search was performed utilising PubMed, Scopus and EMBASE databases from January 2000 to June 2022. The search strategy included a combination of keywords and Medical Subject Headings (MeSH) including “Hybrid Ablation” OR “Convergent Procedure” AND “Atrial Fibrillation. A total of 228 abstracts were screened after duplicates (8) were removed. Following application of predefined inclusion and exclusion criteria 31 articles remained for full text review. Two reviewers (A.E, A.W.S) assessed the eligibility of the selected papers. Discrepancies between the reviewers were adjudicated by the primary author (A.E). References of included articles were crosschecked in search of potentially relevant studies. A total of 16 studies were included in the systematic review and meta-analysis. The search strategy is presented in Additional file 1: Figure S1.

### Definitions, Inclusion and exclusion criteria

Hybrid AF ablation was defined as a combined surgical and endocardial catheter-based approach. The surgical ablation is typically performed with a thorascopy, or a subxiphoid/transdiaphragmatic approach. Surgical lesion sets were defined as any cut/sew lines, radiofrequency or cryoablation performed on the heart through these approaches. Endocardial lesion sets followed the surgical ablation (either concurrently performed or during a staged procedure), and consisted of radiofrequency or cryoablation sets performed via a catheter-based approach. Mid-term was defined as two years or more, and this was arbitrarily chosen as longer term (> 5 year) follow up of hybrid ablation is yet to be reported, wheras 24 month follow up is now being uniformly reported. The inclusion criteria for systematic analysis were (1) patients undergoing hybrid ablation for atrial fibrillation (2) follow up of at least 2 years or median or mean follow up of at least 24 months (3) Freedom from AF reported (4) Mortality and morbidity reported. Case reports, editorials, reviews and preexisting meta-analysis were excluded.

### Primary and secondary endpoints, study quality appraisal

**Fig. 1:**
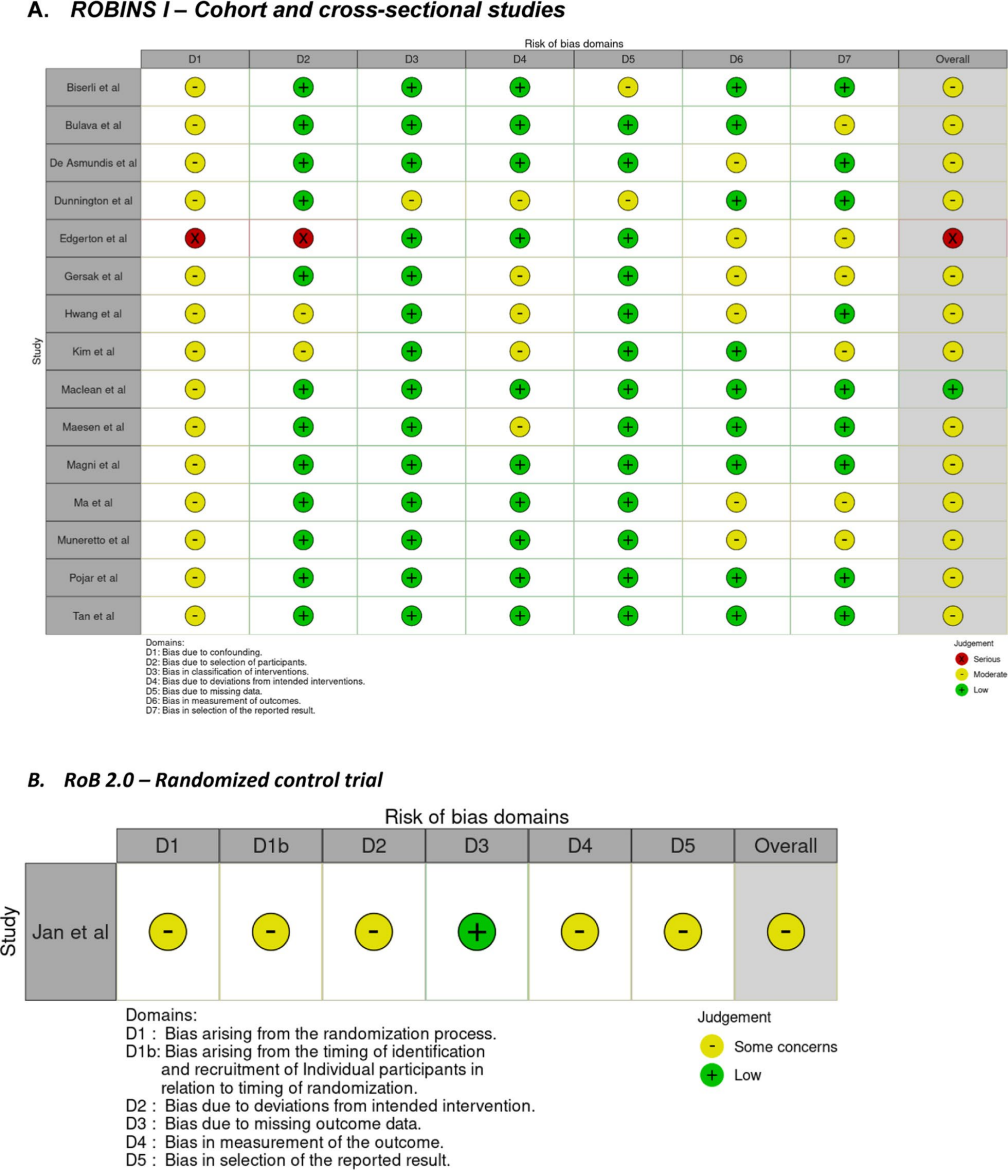
Risk of bias

The primary endpoint for this systematic review was mid-term (at least 2 year) freedom from AF regardless of anti-arrhythmic drugs (AAD) status. The definition of freedom from AF was derived from the individual studies definition. Subgroup analysis was performed assessing freedom from AF off (AAD, by timing (staged vs same sitting), by basic epicardial lesion set (pulmonary vein isolation–PVI vs Box), and by additional ablations (Left atrial appendage–LAA, Ganglionated Plexus–GP and Ligament of Marshall–LoM). Secondary endpoints were death and reported significant complications rates post-procedurally, as defined by the study. These include conversion to sternotomy, esophageal injury, pacemaker implantation, phrenic nerve injury and pericardial effusion, occurring within 30-days of the procedure. Study quality of the included studies was assessed using the Risk of Bias in Non-randomized Studies of Interventions (ROBINS-I) tool for cohort studies, and the Risk of Bias 2 tool (RoB2) for randomized control trials (Fig. 1). [12, 13]

### Data extraction and analysis

All data was extracted to pilot forms and entered into an excel database. For baseline variables, nominal data was recorded as the number of events (n) and expressed as a percentage. Continuous variables were either expressed as a mean and standard deviation (SD) or median and interquartile ranges (IQR). For statistical analysis, medians and IQR were first converted to mean and standard deviation utilising the method outlined by Hozo et al [14]. Baseline patient data is presented in Table 1, and primary and secondary endpoints are presented in Table 2. Statistical analysis was carried out using Stata® (Version 17.0, StataCorp, Texas, USA). A meta-analysis of proportions was performed using the *metaprop* function, with a Freeman-Tukey arcsine transformation. A random effects model was utilised to account for varied study design, experience of the surgeons, centre protocol and population. Results were expressed as forest plots were appropriate, with cumulative proportion expressed as a single percentage. Subgroup analysis was performed by using the *metaprop, by(group)* function, and P < 0.05 denoted statistical significance for intergroup comparison. Heterogeneity was assessed using the I^2^ test statistic. Low heterogeneity was denoted by I^2^ < 50%, moderate heterogeneity by I^2^ 50–74%, and high heterogenei.ty by I^2^ > 75%. Kaplan–Meier survival curves were digitized where numbers at risk were presented, and an algorithmic computational tool was utilized to derive individual patient data as outlined by Guyot et al [15]. Event and censoring data were compiled for 3 years, and overall survival curves were produced with Stata (Version 17.0, StataCorp, Texas, USA) ®.

**Table 1:** Baseline patient variables

Study	Year	Cohort size	Age (years)	Male %	HTN %	BMI (kg/m^2^)	LA diameter (cm)	PAF%	Persistent AF %	Mean Duration (months)	LVEF (%)
Bisleri et al [16]	2012	45	62.3 ± 9.8	33 (73%)	34 (76%)		5.1 ± 0.97	0	45 (100%)	83.8 ± 69.1	56.3 ± 7
Bulava et al [17]	2017	70	62.4 ± 7.9	49 (70%)	55 (79%)	30.8 ± 4.6	150 ± 34 (ml)	0	70 (100%)	41.8 ± 35	64 ± 8
De Asmundis et al [18]	2019	51	65.7 ± 8	34 (67%)	35 (69%)	28.6 ± 4.9	76 ± 20 (ml)	0	51 (100%)	68.3 ± 62.4	56.8 ± 9.8
Dunnington et al [19]	2021	451	67.1 ± 8.4	361 (80%)	NR	30.5 ± 6	4.8 ± 0.8	10 (2%)	441 (98%)	70.8 ± 73.2	51.4 ± 10.5
Edgerton et al [20]	2016	24	63.8 ± 8.9	22 (92%)	15 (62.5%)	30.8 ± 5	5.15 ± 0.28	0	24 (100%)	81.6 ± 42	52.6 ± 8.6
Gersak et al [21]	2016	76	56.6 ± 10.1	NR	NR	NR	4.7 ± 0.5	NR	NR	NR	59.2 ± 12
Hwang et al [22]	2018	72	53.6 ± 8.46	71 (99%)	31 (43%)	25.3 ± 2.6	47.9 ml ± 14	0	72 (100%)	NR	59.1 ± 6.5
Jan et al [23]	2018	50	58.8 ± 6.3	16 (67%)	18 (75%)	30.6 ± 5.9	32.4 ± 8.5 (ml)	25 (100%)	0	49.2 ± 42	65.6 ± 6.15
Kim et al [24]	2021	81	55.4 ± 8.5	80 (99%)	34 (42%)	NR	4.54 ± 0.61	13 (16%)	68 (84%)	NR	NR
Maclean et al [25]	2020	43	68.6 ± 7.7	32 (74%)	NR	NR	4.7 ± 0.63	0	43 (100%)	36 ± 30	50 ± 15
Maesen et al [26]	2018	64	59 ± 9	36 (57%)	44 (68.8%)	26.5 ± 4.7	3.9 ± 1.4	30 (47%)	34 (53%)	59 (12–348) (median/IQR)	55 ± 16
Magni et al [27]	2021	49	57 ± 8.5	44 (90%)	21 (43%)	29 ± 3.5	43.7 ± 10.9 (ml)	0	49 (100%)	34.8 (19.1–65.5) (median/IQR)	52.4 ± 7.5
Ma et al [28]	2021	40	61.4 ± 9.4	30 (75%)	NR	26.8 ± 2.5	4.6 ± 0.46	0	40 (100%)	51.6 ± 31.2	60 ± 7.1
Muneretto et al [29]	2012	36	62.3 ± 10	17 (47%)	15 (42%)	NR	5.0 ± 0.55	0	36 (100%)	72.8 (7–240) (median/IQR)	52.5 ± 3.3
Pojar et al [30]	2019	65	58.2 ± 8.3	49 (75%)	44 (68%)	30.3 ± 4.6	4.5 ± 0.54	30 (46%)	35 (54%)	43 (18.5–79) (median/IQR)	60.5 ± 7.2
Tan et al [31]	2021	50	60 ± 8.2	38 (76%)	NR	26.5 ± 3.5	4.6 ± 0.7	0	50 (100%)	73.3 ± 62.1	62 ± 9.1

**Table 2:** Primary and secondary outcome measures

Study	Year	Follow up duration	Standard dev	# of patients initlally	Freedom from AF	Freedom from AF off AAD	Death	Emergent reopening	Phrenic nerve palsy	Stroke	Major complications
Bisleri et al [16]	2012	28.4(mean)	1.7	45	40/45 (88.9%)	NR	0	0	0	0	0
Bulava et al [17]	2017	24 (final)	0	70	64/67 (95.5%)	NR	0	2/70	7/10	0	9/70 (12.8%)
De Asmundis et al [18]	2019	24.9 (mean)	11.8	51	35/51 (68.6%)	35/51 (68.6%)	0	0	0	0	6/51 (11.8%)
Dunnington G. et al [19]	2021	36 (final)	0	451	104/145 (72%)	96/145 (66%)	7/461	4/455	6/455	2	28/461 (6.1%)
Edgerton et al [20]	2016	24 (final)	0	24	4/21 (19%)	NR	3/24	0/24	1/24	2	5/24
Gersak et al [21]	2016	48 months (final)	0	76	18/28 (64%)	25/36 (69%)	2/76	0	0	0	9/76 (11.8%)
Hwang et al [22]	2018	25.2 (median)	IQR 1.2–3.2	72	43/61 (70.4%)	NR	0	0	0	0	0
Jan et al [23]	2018	30.5 (mean)	6.9	50	19/25 (76%)	14/25 (58.3%)	0	1/24	0	0	3/24
Kim et al [24]	2021	24 months (final)	0	81	55/81 (67.8%)	NR	0	0	0	0	7/81
Maclean et al [25]	2020	30.5 (mean)	13.3	43	25/43 (58.1%)	14/43 (32.6%)	0	1/43	1/43	0	5/43
Maesen et al [26]	2018	36 months (final)	0	64	50/63 (79%)	44/57 (77%)	0	0	0	0	3/63
Magni et al [27]	2021	24 (final)	0	49	33/49 (67%)	NR	0	1/49	2/49	0	2/49
Ma et al [28]	2021	26 (mean)	13.5	40	34/40 (85%)	NR	0	0	0	0	0
Muneretto et al [29]	2012	30 (mean)	NR	36	33/36 (91.6%)	28/36 (77.7%)	0	0	0	0	0
Pojar et al [30]	2019	24 months	0	65	57/65 (87.7%)	NR	0	0	1/65	0	1/65
Tan et al [31]	2021	29 (mean)	12	50	35/50 (70%)	35/50 (70%)	0	0	0	0	6/50

### Assessment of bias and heterogeneity

Publication bias was assessed through visual inspection of funnel plots and Begg’s rank correlation test in Stata®. A trim and fill analysis was performed in instances of publication bias. An influential study analysis with adjusted effect sizes computed after the omission of each study. In order to assess the impact of study age (publication year) on effect size, a meta regression was performed comparing year of publication utilising a random effects model. A coefficient n was calculated to assess correlation and P value, with P < 0.05 denoting significance. These results are represented in Additional file 1: Figure S2 and S3. Finally, A subgroup analysis was performed based on the definition of recurrence. As the majority of studies defined recurrence as AF duration greater than 30 s, the groups were divided into “30 s” and “other” (Additional file 1: Figure S4).

## Results

### Baseline study characteristics

The search strategy revealed a total of 235 studies with 8 duplicates, one additional reference was identified on screening included study reference lists, thus a total of 228 studies were screened. After full review, 16 studies with 1242 patients were included in the systematic review [16–31]. The majority of papers were retrospective cohort studies (15) and one study was a randomized control trial (RCT). The cohort sizes ranged from 24 to 451 patients, with the majority of studies reporting relatively small cohort sizes (30–80 patients). The quality of included studies ranged from poor to good as per the ROBINS-I and RoB2 tools, with the majority (14) studies scoring “moderate. The mean follow up was 31.5 ± 8.4 months. The pooled mean age of patients was 62.3 ± 9.7 years, and the pooled BMI was 29.3 ± 5.4. A total of 346 (59%) of patients had hypertension and 912 (73%) were male. The mean left atrial (LA) diameter was 4.69 ± 0.82 cm, and mean LA volume was 66 ± 34 ml. The mean duration of AF (since diagnosis) was 67.7 ± 72 months with a mean LVEF of 55.4 ± 11%. Five studies included patients with paroxysmal AF, with the majority of studies reviewing patients with persistent and long-standing AF [19, 23, 24, 26, 30].

### Characteristics of epicardial (surgical) ablation

The pooled epicardial (surgical) component data showed a mean procedural time of 77–222 min. The predominant lesion was a box lesion (12 studies), with 4 studies performing epicardial pulmonary vein isolation [21, 23, 25, 26]. The majority of studies utilized bipolar radiofrequency epicardial ablation, however four studies utilized unipolar energy [16]^.^ [20, 21, 29]. The most common access was thorascopic (12 studies). Seven studies utilised a bilateral thorascopic approach [17, 19, 22, 24, 26, 27, 30]. The convergent procedure was performed in four studies via a subxiphoid laparoscopic approach [20, 21, 23, 25]. Surgical Left atrial appendage (LAA) exclusion was variably performed, performed across all patients in 6 studies [18–20, 24, 28, 31]. Ganglionated plexus (GP) ablations were performed in 7 studies [17–19, 22, 24, 28, 31]. Ligament of Marshall ligations were performed in 8 studies (with one study performing it in 67 of 72 patients) [17–19, 22, 24, 28, 30, 31]. These results are summarised in Additional file 1: Table S2.

### Characteristics of catheter based (endocardial) ablation

Procedural data for the endocardial component are summarised in Additional file 1: Table S3. Six studies conducted endocardial ablation in staged setting, whereas two studies had mixed cohorts [16, 17, 19, 21, 22, 24, 25, 29, 30]. The remaining studies performed endocardial ablation during the same procedure. Additional linear ablation lines were performed in 13 studies wheras three studies did not conduct any further linear ablation [21, 23, 31]. Eleven studies performed additional cavotricuspid isthmus (CTI) ablations, five studies performed complex fractionated atrial electrocardiograms (CFAE) ablation and seven studies performed a linear ablation to the mitral isthmus. Additional ablations were performed in 11 studies for recurrence [16, 18–21, 23, 25–29]. Twelve studies utilised electro-anatomical mapping with the majority utilising CARTO (Biosense Webster, Diamond Bar, CA, USA), whereas the four studies did not specify their technique of mapping. The most common modality of post-procedural monitoring was Holter (ranging from 24 h to 7 days), whereas internal loop recorders (ILRs) were utilised in 5 studies [16, 19, 21, 23, 29]. The definition of freedom from AF varied across studies, with studies most commonly defining recurrence as “documented episodes of tachyarrhythmias lasting > 30 s” (12 studies). Most studies incorporated a blanking period following endocardial ablation, however three did not [16, 17, 29].

### Primary endpoint

**Fig. 2:**
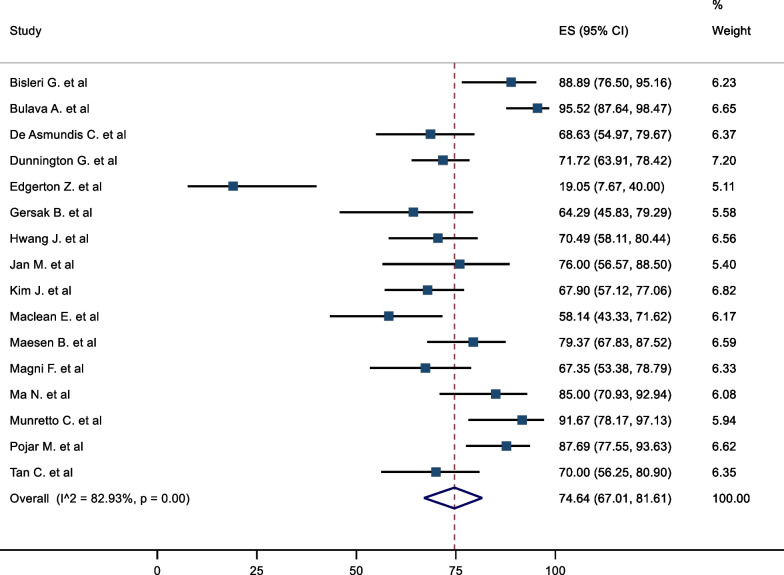
Mid-Term Freedom from AF

**Fig. 3:**
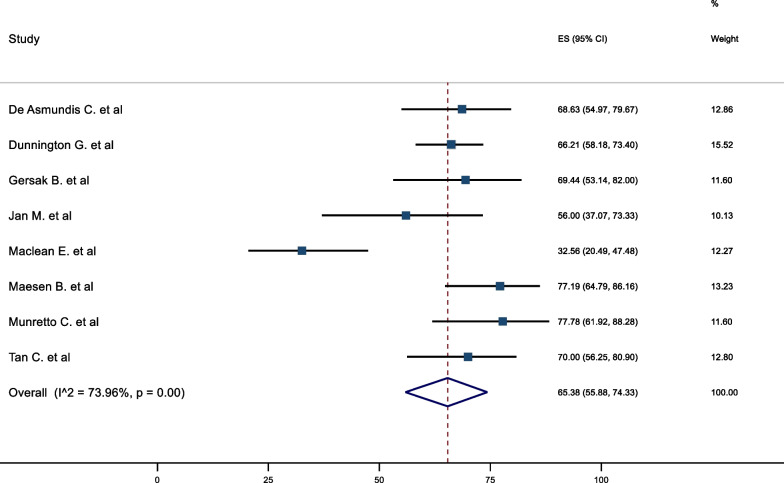
Mid-Term Freedom from AF off AAD

**Fig. 4:**
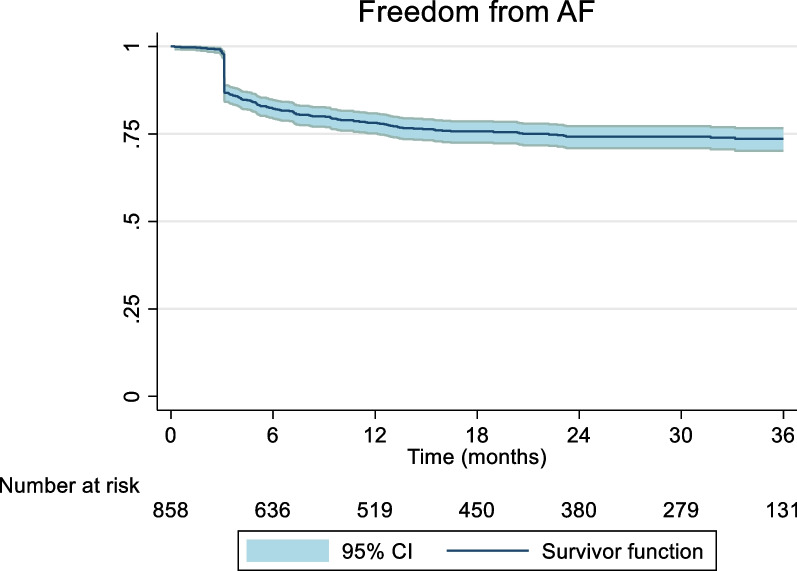
Survival curve, freedom from AF

The mid-term freedom from AF was 74.64% (95%CI 67.01–81.61). There was significant heterogeneity associated with the result, (I^2^ = 82.93%) (Fig. 2). A meta-regression analysis was conducted to assess the impact of left ventricular ejection fraction (LVEF), LA size, duration AF and BMI on freedom from AF to further explain the potential sources of heterogeneity. None of these results demonstrated a statistically significant association. The corresponding mid-term freedom from AF off class 1 and 3 AAD was 65.38% (95%CI 55.88–74.33). This result was associated with moderate heterogeneity (I^2^ = 73.96%) (Fig. 3). Nine studies presented survival curves with numbers at risk, appropriate for aggregation. The aggregate AF free survival at time points 6, 12, 18, 24, 30 and 36 months was 82.2%, 78.2%, 75.7%, 74.2%, 74.2% and 73.6% respectively (Fig. 4).

### Subgroup analysis

**Fig. 5:**
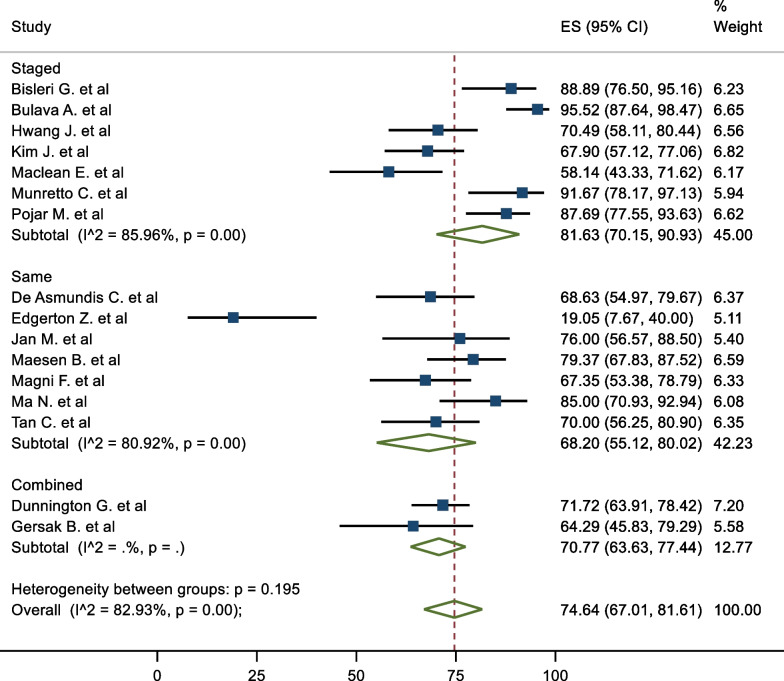
Mid-Term Freedom from AF by Staged/Same sitting procedure

**Fig. 6:**
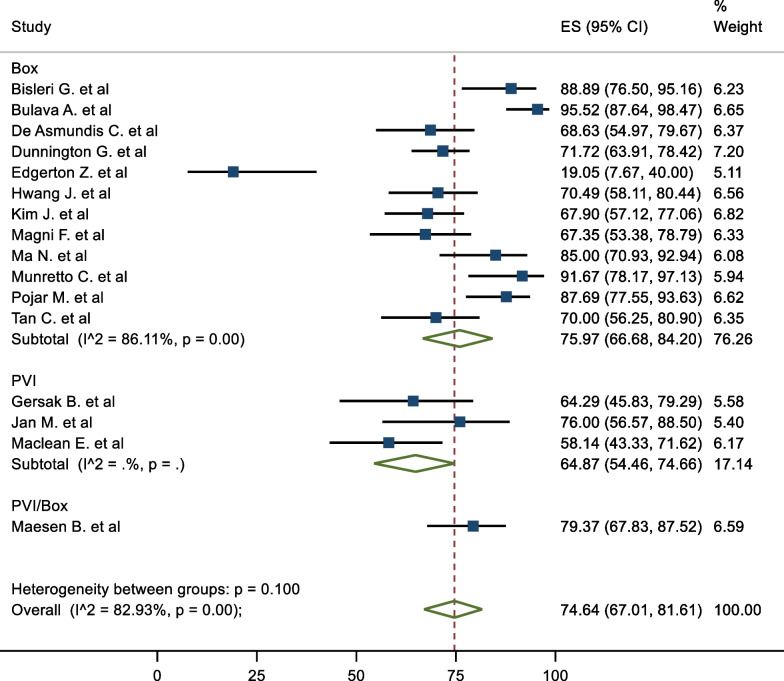
Mid-Term Freedom from AF by epicardial lesion set

**Fig. 7:**
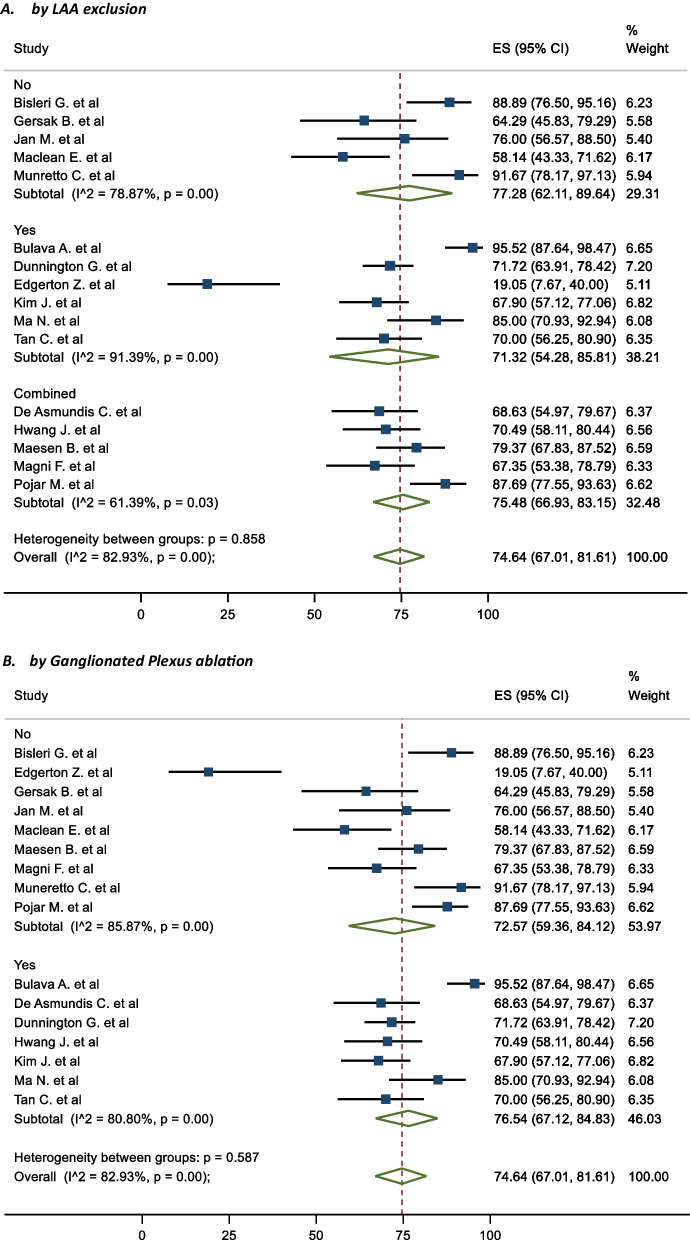

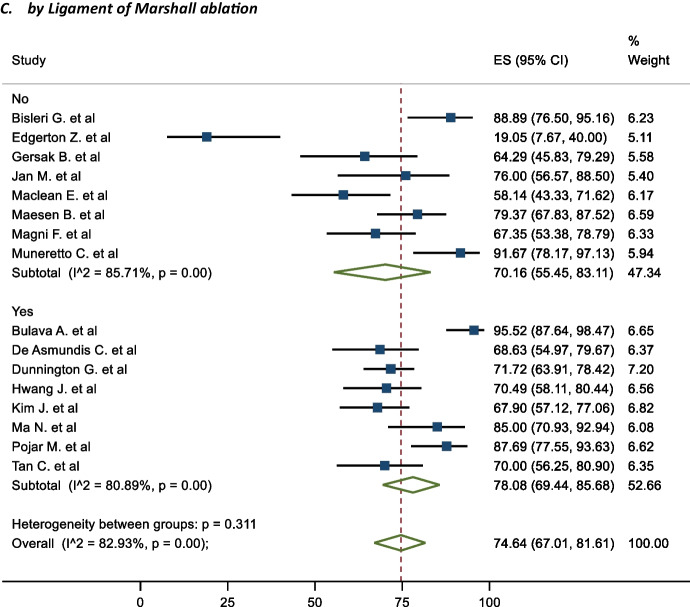
Mid-Term Freedom from AF

**Fig. 8:**
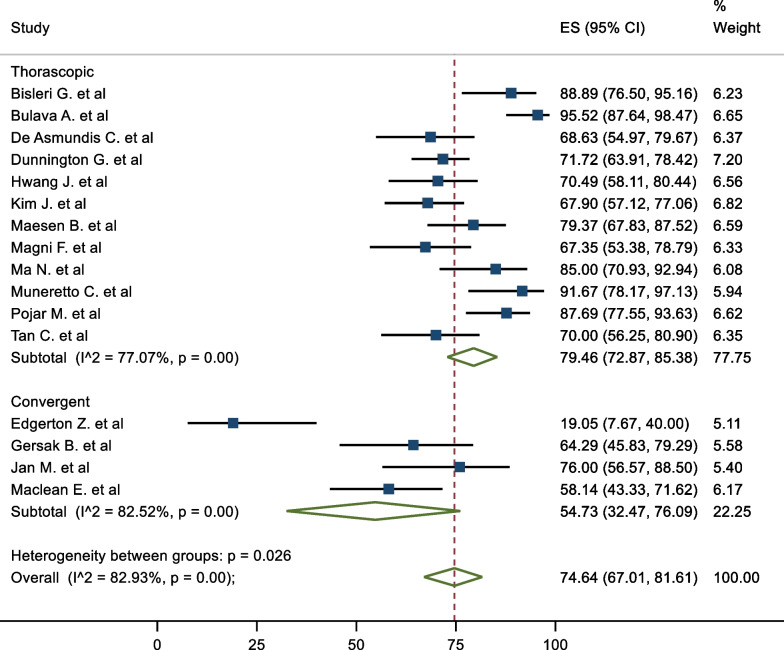
Mid-Term Freedom from AF by access

In terms of subgroups, the mid-term freedom from AF of staged procedures was 81.63%, and same sitting procedures was 68.20% (Fig. 5). There were no significant differences between the cohorts (P = 0.195). The corresponding mid-term FFAF based on epicardial lesion set was 75.97% and 64.87% for box and PVI respectively (Fig. 6). This did not reach statistical significance (P = 0.100). One study performed predominantly a PVI with a completion of a box lesion if AF was not terminated [29]. The Mid-term freedom from AF based on LAA exclusion, GP or LoM ablation did not demonstrate a significant difference between the cohorts (71.32% vs 77.28%, 72.57% vs 76.54% and 70.16% vs 78.08% respectively) (Fig. 7). The midterm freedom from AF based on procedure access (thorascopic vs subxiphoid convergent) demonstrated a statistically significant result, with a freedom from AF of 79.46% and 54.73% in the thorascopic and convergent cohorts respectively (P = 0.026) (Fig. 8).

### Secondary endpoint

**Fig. 9:**
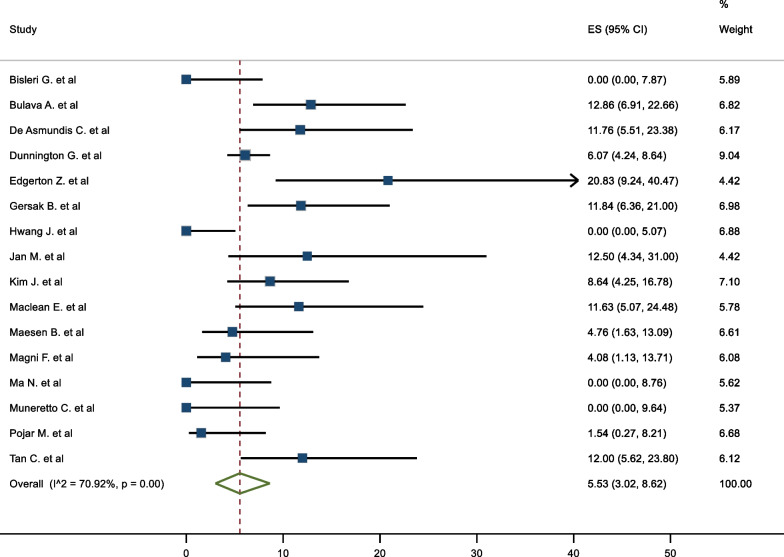
Complications

All studies reported secondary endpoints of mortality and morbidity. The rate of post procedural complications ranged from 0% to 20.8%, with a pooled complication rate of 5.53% (95%CI 3.02–8.62%). There was moderate heterogeneity in the results (I^2^ = 70.92%). (Fig. 9). Phrenic nerve palsy was observed in 18 patients (1.44%). A total of 4 (0.32%) patients sustained a postoperative stroke. Nine patients (0.72%) required sternotomy for bleeding. Three studies had reported mortalities, with a total of 12 deaths (0.97%) overall. Only four deaths were a direct mechanical complication of the procedure (atrio-esophageal fistulae). Two patients died of a stroke. No patients died as a result of bleeding or cardiac perforations. 10 of the 12 deaths occurred within 30 days of the procedure (Table 2).

### Assessment of Bias

**Fig. 10:**
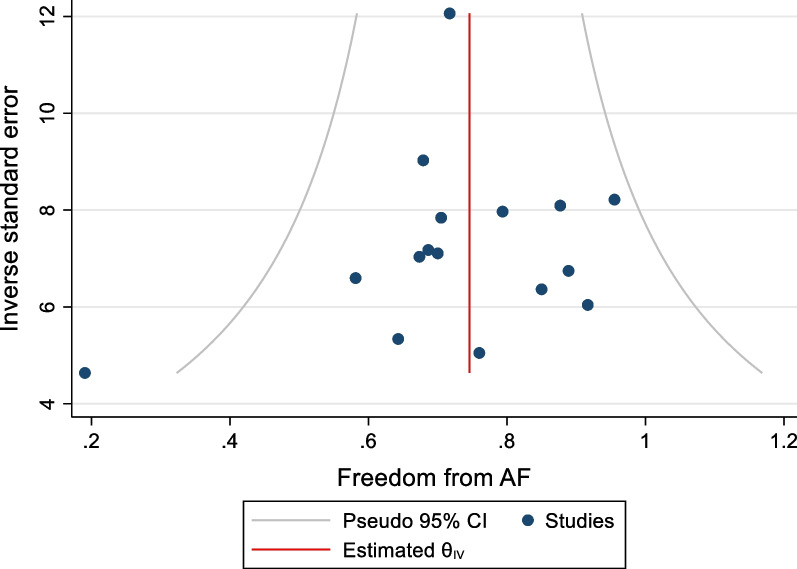
Funnel plot of FFAF

**Fig. 11:**
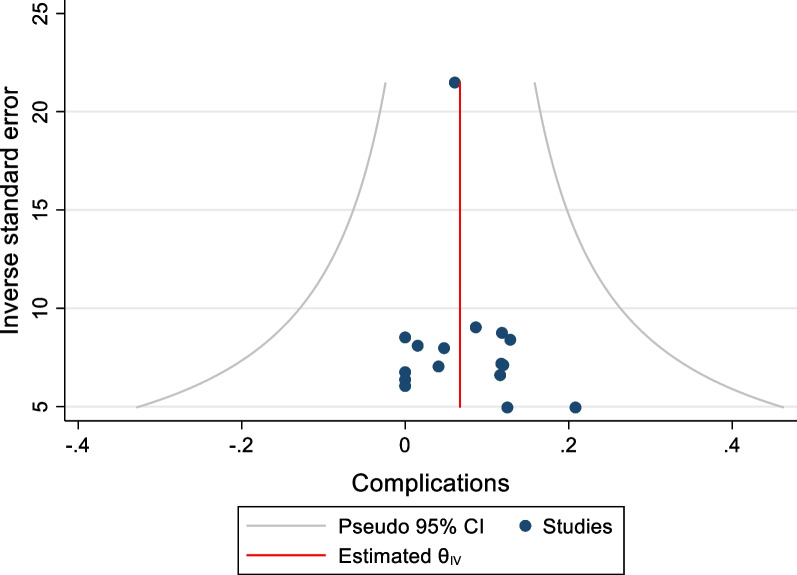
Funnel plot of complications

There was no evidence of publication bias on visual inspection of funnel plots of both freedom from AF and complications (Figs. 10 and 11). Beggs rank test produced a statistically insignificant result in both outcomes. Meta regression analyses comparing year and study and study size to freedom from AF did not demonstrate a significant relationship between the two variables (coeff n = − 0.16, P = 0.194). A leave one out analysis highlighted the potential effects of two studies [17, 20]. As such, the omission of Edgerton et al. demonstrated an increased the mid-term freedom from AF to 76% (95%CI 69–83%), and the omission of Bulava et al. decreased the mid-term freedom from AF to 73% (95%CI 66–80%). Neither of these studies impacted the significance of the effect size (P < 0.01 pre and post omission). Finally, there was still significant heterogeneity despite categorizing freedom from AF based on definition of recurrence.

## Discussion

Hybrid approaches for AF ablation are an emerging field and are now being offered to a wider patient population. *Varzaly *et al. systematically assessed hybrid ablation and reported favorable short-term (19 month) sinus rhythm maintenance (SRM) of 79% over 22 studies and 925 patients [10]. A number of cohort studies and randomized control trials have been published since, reporting mid-term results [18, 19, 23–25, 31]. This systematic review demonstrates four key findings. Firstly, A mid-term (mean follow up of 31.5 months) freedom from AF of 74.6% and 65.4% off AAD. Secondly, an actuarial freedom from AF of 78.2%, 74.2% and 73.6% at 1, 2 and 3 years respectively. Thirdly, there were oo significant differences in mid-term freedom from AF based epicardial lesion set (box vs PVI) or LAA/GP/LoM ablation or staged vs concomitant procedures. Lastly, complication rate was low at 5.53%. The long-term outcomes following ablation provides practical information on the effectiveness of the procedure. The CM4 procedure is the ‘gold standard’ for SR maintenance, with a 5-year and 10-year freedom from atrial tachyarrhythmia of 84% and 77% respectively [6]. Ganesan et al. report the 3-year freedom from AF in a similar cohort of patients undergoing catheter ablation at 41.6% and 77.8% following a single procedure and multiple procedures respectively [4]. Five-year outcomes follow catheter ablation have demonstrated further attrition in freedom from AF [32]. As hybrid ablation is an evolving field, long term follow up is not yet available and future studies will provide further insight into the effectiveness of the procedure.

Hybrid ablation may result in superior freedom from AF when compared to catheter ablation in the setting of persistent AF substrates. Pulmonary vein isolation through an endocardial approach remains the mainstay of AF ablation in patients with paroxysmal AF, as the foci of arrythmia remain the pulmonary veins and antrum. As AF goes from paroxysmal to persistent, it becomes less of a focal disease and more anatomically diffuse [33]. The left atrium becomes heterogenous and infiltrated with scar. Furthermore, the junction between the pulmonary veins and left atrium is less of an initiator and the left atrium becomes an independent source of arrythmias [33]. The advantage of the hybrid approach is the ability to isolate the posterior left atrial wall, exclude the appendage, map and validate lesions as well as perform further linear ablations with precise electrophysiological (EP) endpoints. This ensures transmural lesions, which is potential limitation of thorascopic ablation or catheter ablation in isolation [34]. Hybrid ablation has demonstrated superior outcomes when compared to catheter ablation in literature. Van der Heijden summarised 34 papers on hybrid and catheter ablation in patients with longstanding AF, demonstrating a significantly higher freedom from AF (70.7% vs 49.9%, P < 0.001) [11]. Within this systematic review, four papers compared hybrid ablation to RFCA, three of which studied patients with longstanding AF, one studied paroxysmal AF. Of the four, three papers demonstrated a significantly higher mid-term freedom from AF in the hybrid cohorts. The results of future RCT’s comparing hybrid ablation to catheter ablation may further validate the role of hybrid ablation in longstanding AF [35].

This study provides important technical insights. Firstly, there were no significant differences in the mid-term freedom from AF between studies that performed a staged or same sitting procedure. Advantages of a sequential approach is the immediate identification of lesion gaps that can be corrected by catheter ablation and shorter procedural times [10]. Advantages of a staged approach is that it allows time for lesions to mature and edema to regress, identifying definite lesions for further endocardial ablation [10]. Logistical issues regarding availability of cardiothoracic and cardiology teams also influences the approach. Varzaly et al. performed a similar subgroup analysis in their meta-analysis, and also did not find a significant difference between the two cohorts [10]. At this stage, there is no compelling argument towards either approach.

Secondly, we report a lower mid-term freedom from AF in patients undergoing the convergent procedure, compared to a unilateral or bilateral thorascopic approach. The convergent procedure was designed as closed chest hybrid approach, utilising a subxiphoid incision to directly access the posterior left atrium. The latest iteration of the procedure creates broad, epicardial linear lesions with the intention to homogenize the posterior LA wall [36]. A vacuum assisted RF ablation catheter is used to suction atrial tissue into apposition with the RF coil, ensuring that energy is delivered towards the posterior LA wall and away from the esophagus [36]. This is followed by an endocardial ablation to confirm lesion integrity and supplement the epicardial procedure. One explanation for the inferior freedom from AF is that posterior LA ablation is limited in the subxiphoid approach as it is confined within the boundaries of the oblique sinus. Convergent ablation is also in its formative stages and early studies are prone to operator bias. One such study utilised unipolar radiofrequency devices and had small operator numbers, potentially accounting for its poor outcomes [20].

Thirdly, there were no significant differences in mid-term freedom from AF based on LAA exclusion, GP ablation or LOM ligation. This result is surprising, as LAA exclusion potentially improves freedom from AF, in light of recent evidence from systematic reviews and randomized catheter ablation data [10, 37]. The caveat of the BELIEF trial was that isolation was performed electrically [37]. A further caveat of this study was that approximately a third of patients undergoing standard ablation alone underwent LAA ligation as they had AF recurrence [37]. The aMAZE trial compared PVI alone to LAA ligation and PVI utilising the Lariat device, which is more akin to a surgical isolation [38]. This was performed in a population of patients with longstanding AF that failed AAD [38]. There was no significant difference in AF recurrence between the groups [38]. Further research into the role of LAA exclusion in hybrid surgery is required. GP ligation is more contentious. Randomized data from thorascopic cohorts do not demonstrate a benefit in persistent AF and are associated with a potentially larger number of complications [39]. Animal studies demonstrate that despite ablation, re-innervation occurs over time which may explain its ineffectiveness [40].

A final technical consideration is the choice of epicardial lesion sets. The majority of studies employed a box or wide antral lesion set, with pulmonary vein isolation and a roof/floor line connecting the pulmonary veins. This study did not find a statistically significant difference between the two approaches. The main concern of pulmonary vein isolation, especially in the setting of persistent AF, is pulmonary vein reconnection [41]. This is reflected in literature; studies assessing endocardial lesion sets demonstrate a greater freedom from AF when a wide antral or box lesion set is employed as opposed to a pulmonary vein isolation alone [42]. The same lesion sets assessed in surgical patients demonstrate a greater freedom from AF when a biatrial maze is performed as opposed to PVI alone, however these results are underpowered [43]. High quality and randomized data of hybrid cohorts are well poised to address these all these technical considerations.

The incidence of major complications was 5.53%, which is similar to previous meta analysis [10, 44]. There was a large degree of heterogeneity, perhaps reflective of the experience of the variance centers. For instance, three studies reported a complication rate of 0, whereas one centre reported a complication rate of 20%. This particular centre had small patient numbers and reported hybrid intervention as a “first”, thus the study being prone to design bias. There were only 12 deaths overall, three of which were reported in the aforementioned study. As Hybrid AF ablation is still a novel procedure, the complication rate is expected to decrease with further experience and iterations. For example, Bulava et al. modified the surgical technique to minimize the rate of phrenic nerve palsy [17]. Furthermore, De Lurgio et al. recommended prescribing NSAID therapy following convergent ablation, to mitigate the risk of inflammatory pericardial effusions [45]. The use of bipolar radiofrequency devices have replaced monopolar devices are more infrequent due to mitigate the risk of atrioesophageal fistulae [10].

There are a number of important limitations. Firstly, the absence of individual patient data limiting subgroup analysis based on preoperative patient characteristics. Variable preoperative left atrial sizes, duration of AF and proportion of patients with longstanding and persistent AF may have influenced the procedural effectiveness. A second limitation was significant heterogeneity between centers within the study. There was variable operator experience, epicardial and endocardial lesion sets, different ablation devices varying rates of follow up and different definitions of “freedom from AF’. We opted to quantify this by performing a subgroup analysis based on the definition of recurrence, however there was still significant resultant heterogeneity. Other sources of heterogeneity include the different baseline characteristics of patients and age of the study, whereby older generation ablation catheters and mapping technology was used. Thirdly was a significant loss of follow up over time, with some studies reporting more than half being lost to follow up [19]. This introduces a potential “healthy patient bias”. Fourthly, the mean age of patients was 62 years old with largely longstanding atrial fibrillation, and the extrapolation of this cohort to the general population may be inaccurate. Lastly the overall strength of evidence was average, with the majority of studies being cohort designs. The introduction of cohort designs creates selection bias. For example, Edgerton et al. demonstrated a significant deviation in terms of complications and freedom from AF. The study was designed such that patients who refused hybrid ablation underwent catheter ablation, therefore introducing a potential bias [20]. The recent CONVERGE trial sought to minimize these limitations by randomizing patients to hybrid ablation in a 2:1 fashion [45]. Future randomized studies such as the HARTCAP -AF will better control for these limitations.

## Conclusion

Hybrid ablation demonstrates favorable mid-term freedom from AF with an overall low complication rate. There are no significant differences in effectiveness based on staging of procedure or removing/ablating the LAA, LoM or GOP. A thorascopic hybrid approach may be more effective than convergent ablation. Current outcomes are limited to largely retrospective cohort studies with some randomized data being recently published. Further analysis of high-quality studies with randomised data and long-term follow up will help verify these results.

## Supplementary Information

**Additional file 1.** Supplementary Figures and Tables.

## Data Availability

The datasets used and/or analysed during the current study are available from the corresponding author on reasonable request.
