# Supplementary material for: Mid term freedom from atrial fibrillation following hybrid ablation, a systematic review and meta analysis
Source: J Cardiothorac Surg. 2023 Apr 19;18:155. doi: 10.1186/s13019-023-02189-2 (PMC10114378; doi:10.1186/s13019-023-02189-2)
Supplement: Supplementary file 1 — Additional file 1. Supplementary Figures and Tables. [file 13019_2023_2189_MOESM1_ESM.docx]

Records excluded

N = 197

Full-text articles assessed for eligibility
n = 31

Records screened
n = 228

Records after duplicates removed
n = 228

## Identification

Records Identified through Database Searches
n = 235

Additional records identified through reference check

N = 1

## Screening

## Eligibility

Reasons for exclusion (15)

Follow up < 24 months (9)

Wrong study design (3)

Duplicated population (2)

Concurrent cardiac surgery (1)

## Included

Studies included in qualitative synthesis

N = 16

**Supplementary Figure 1.** PRISMA flow-chart summarizing the search strategy for relevant publication

***Supplementary Figure 2: Leave one out analysis***

’

***Supplementary figure 3: Meta regression of FFAF and year***

***Supplementary figure 4: FFAF by follow up***

| Supplementary Table 1: Surgical Characteristics | | | | | | | | | |
| --- | --- | --- | --- | --- | --- | --- | --- | --- | --- |
| Study | Surgical Time | Basic lesion | Device | Mapping | Energy | Access | LAA exclusion | Ganglionated Plexus | Ligament of Marshall |
| Bisleri G. et al | NR | Box | COBRA | CARTO | Radiofrequency Unipolar | Thorascopic (right) | No | No | No |
| Bulava A. et al | 222 ± 48 | Box | Atricure | CARTO | Radiofrequency Bipolar | Thorascopic  (bilateral) | Some (NR) | Yes | Yes |
| De Asmundis C. et al | Total  174.8±91.7 | Box | Atricure | CARTO | Radiofrequency Bipolar | Thorascopic  (left) | Yes | Yes | Yes |
| Dunnington G. et al | NR | Box | Atricure 44%  Atricure + COBRA 56% | CARTO | Radiofrequency Bipolar | Thorascopic  (bilateral) | Yes | Yes | Yes |
| Edgerton Z. et al | 175.15±67.34 | Box | nContact | NR | Radiofrequency Unipolar | Convergent (subxiphoid) | Yes | No | No |
| Gersak B. et al | NR | PVI | EpiSense  (atricure) | NR | Radiofrequency Unipolar | Convergent (subxiphoid) | No | No | No |
| Hwang J. et al | NR | Box | Atricure | CARTO | Radiofrequency Bipolar | Thorascopic  (bilateral) | Some (69/72) | Yes | Some (67/72) |
| Jan M. et al | 77±29 | PVI | EpiSense (atricure) | NR | Radiofrequency Bipolar | Convergent  (subxiphoid) | No | No | No |
| Kim J. et al | NR | Box | Atricure | CARTO | Radiofrequency Bipolar | Thorascopic  (bilateral) | Yes | Yes | Yes |
| Maclean E. et al | NR | PVI | EpiSense  (atricure) | CARTO | Radiofrequency Bipolar | Convergent  (subxiphoid) | No | No | No |
| Maesen B. et al | NR | PVI ± Box | Atricure | NR | Radiofrequency Bipolar | Thorascopic  (bilateral) | Some (23/64) | No | No |
| Magni F. et al | 204±77 | Box | Atricure | EnSITE | Radiofrequency Bipolar | Thorascopic  (bilateral) | Some (16/49) | No | No |
| Ma N. et al | NR | Box | Atricure | CARTO | Radiofrequency Bipolar | Thorascopic  (left) | Yes | Yes | Yes |
| Muneretto C. et al | 80±7 | Box | COBRA | CARTO | Radiofrequency Unipolar | Thorascopic  (right) | No | No | No |
| Pojar M. et al | Total  120.5±22 | Box | Gemeni s (Medtronic) | CARTO | Radiofrequency Bipolar | Thorascopic  (bilateral) | Some (22/65) | No | Yes |
| Tan C. et al | 175±34.9 | Box | Atricure | CARTO | Radiofrequency Bipolar | Thorascopic  (left) | Yes | Yes | Yes |

| Supplementary table 2: Endocardial ablation characteristics. | | | | | | | |  |
| --- | --- | --- | --- | --- | --- | --- | --- | --- |
| Study | Time | All patients endocardial? | Linear Ablation Lines | Staged | subsequent ablations | Monitoring | Definition of freedom from AF | Blanking Period |
| Bisleri G. et al | NR | Yes | CFAE (44%), CTI (24%), PV (7%) | Staged | Yes | ILR | Absence of AF durations < 5min, Overall AF burden of 0.5% per month | No |
| Bulava A. et al | 131 ± 38 | Yes | Posterior LA wall, MI line, CTI line | Staged | No | 7-day holter | All documented episodes of atrial tachyarrhythmias lasting ≥ 30 s | No |
| De Asmundis C. et al | Total  174.8±91.7 | Yes | CTI + MI line + CFAE, | Same | Yes 6/51 | 24H holter | All documented episodes of atrial tachyarrhythmias lasting ≥ 30 s | Yes – 3 months |
| Dunnington G. et al | NR | Yes | MI line, CTI line | 45 Same - 410 Staged | Yes 34/451 | ILR | All documented episodes of atrial tachyarrhythmias lasting ≥ 30 s | Yes – 3 months |
| Edgerton Z. et al | 101.31±23.24 | Yes | CS, LAA, CFAE, | Same | Yes | 7-day holter | Any episode of atrial fibrillation/atrial tachycardia longer than 30 seconds | Yes – 12 weeks |
| Gersak B. et al | NR | Yes | No | 60 - same, 16 staged | Yes | ILR | AF/AFL/AT with an AFB less than 3% continuously through a minimum of 36 months follow-up and off class I and III AADs | Yes – 3 months |
| Hwang J. et al | NR | Yes | SVC, CTI, MI line | Staged | No | 24H holter | All documented episodes of atrial tachyarrhythmias lasting ≥ 30 s | Yes – 3 months |
| Jan M. et al | 147±35 | Yes | No | Same | Yes 4/25 | ILR | Overall AF burden of 1% | Yes – 3 months |
| Kim J. et al | NR | Yes | CTI, SVC, Septal line, perimitral line | Staged | No | 24H holter | All documented episodes of atrial tachyarrhythmias lasting ≥ 30 s | Yes – 3 months |
| Maclean E. et al | NR | Yes | CFAE, otherwise not reported | Staged | Yes 11/43 | 72H holter | All documented episodes of atrial tachyarrhythmias lasting ≥ 30 s | Yes – 3 months |
| Maesen B. et al | NR | Yes | MI line, CTI, CFAE | Same | Yes 10/64 | 7-day holter and 24H holter | All documented episodes of atrial tachyarrhythmias lasting ≥ 30 s | Yes – 3 months |
| Magni F. et al | 117±34 | Yes | CTI | Same | Yes 10/49 | 72H holter | All documented episodes of atrial tachyarrhythmias lasting ≥ 30 s | Yes – 90 day |
| Ma N. et al | NR | Yes | CTI, MI line | Same | Yes 2/40 | 24H holter | All documented episodes of atrial tachyarrhythmias lasting ≥ 30 s | Yes – 3 months |
| Muneretto C. et al | 18±12 | No (22/36) | CTI, CFAE | Staged | Yes | ILR | Absence of AF durations < 5min, Overall AF burden of 0.5% per month | No |
| Pojar M. et al | Total  120.5±22 | Yes | CTI | Staged | No | 24H holter | All documented episodes of atrial tachyarrhythmias lasting ≥ 30 s | Yes – 3 months |
| Tan C. et al | 163.7±48.1 | Yes | No | Same | No | 24H holter | All documented episodes of atrial tachyarrhythmias lasting ≥ 30 s | Yes – 3 months |

| **Footnote for supplementary tables**  LAA: Left atrial appendage  CFAE: complex fractionated atrial electrogram  LA: left atrial  CTI: Cavotricuspid isthmus  MI line: Mitral isthmus line  CS: Coronary sinus  SVC: Superior vena cava  ILR: Internal loop recorder |
| --- |
